# Deconstructing the Master Switch: Advances in Direct NLRP3 Inhibition

**DOI:** 10.3390/ph19071104

**Published:** 2026-07-17

**Authors:** Yiming Xu, Sasha Murphy

**Affiliations:** Department of Medicinal Chemistry, School of Pharmacy, Virginia Commonwealth University, Richmond, VA 23298, USA; murphysa3@vcu.edu

**Keywords:** direct NLRP3 inhibitor, NLRP3 inflammasome activation, crystallization structures, NACHT domain

## Abstract

As a bona fide “master switch,” NOD-like receptor family pyrin domain containing 3 (NLRP3) functions not only as an inflammatory mediator but also as a primary sensor of metabolic stress and danger signals. Its dysregulation has been implicated in an exceptionally broad spectrum of human diseases, making it one of the most intensively studied therapeutic targets. While the discovery of the first direct antagonist MCC950 marked a turning point, the subsequent explosion of diverse inhibitor classes demands a systematic, up-to-date evaluation. This review comprehensively analyzes the current landscape of direct NLRP3 inhibitors, focusing on how recent structural breakthroughs illuminate specific mechanism-of-action differences. We systematically reviewed peer-reviewed literature from 2015 to 2026, categorizing small-molecule inhibitors based on their chemical scaffolds and binding pockets as revealed by cryo-EM and X-ray crystallography data. By mapping these structural insights into functional outcomes, we provide a definitive molecular-level analysis designed to guide the rational design and optimization of next-generation NLRP3-targeted therapeutics.

## 1. Introduction

The NOD-like receptor family pyrin domain containing 3 (NLRP3) protein is a critical component of the innate immune system, responsible for sensing danger signals and pathogens through damage-associated molecular patterns (DAMPs) and pathogen-associated molecular patterns (PAMPs) [1,2,3]. Once stimulated, it can form multimeric protein complexes with an adaptor, apoptosis-associated speck-like protein (ASC), and an effector, pro-caspase-1, to form a multimeric protein termed the “NLRP3 inflammasome” [4]. The subsequent inflammatory responses trigger the release of pro-inflammatory cytokines interleukin (IL)-1β and IL-18, mediated by caspase-1 activation and gasdermin D (GSDMD) cleavage to maintain homeostasis [5,6,7].

The transient activation of NLRP3 is protective, highly conserved and spatiotemporally regulated, acting as the rapid, self-limiting alarm to neutralize pathogens and eliminate threats to prevent chronic tissue damage [8,9,10,11]. In contrast, prolonged or chronic activation of NLRP3 leads to pathological inflammation and tissue damage, contributing to the pathogenesis of various diseases, including neurodegenerative, metabolic, cardiovascular and autoimmune disease [12,13,14,15,16,17,18,19].

NLRP3 has become a premier therapeutic target due to its “master switch” role in inflammation. Targeting NLRP3 directly provides a superior pharmacological advantage over downstream cytokine blockers [20,21]. By limiting excessive tissue damage while preserving core innate immune functions, selective NLRP3 inhibition avoids the widespread immunosuppression typical of broader therapies. Consequently, direct inhibitors represent a highly specific, potent strategy to manage chronic autoinflammatory, metabolic, and neurodegenerative diseases [22,23].

In this review, we focus on the method for hit screening, structural insights into the binding modes of NLRP3 inhibitors, and modification strategies, aiming to facilitate the development of novel therapeutics.

## 2. Structural Insight into NLRP3 Activation

Methodology Note: This study utilizes a systematic, comparative structural bioinformatic review design to evaluate the conformational transitions of the full-length NLRP3 protein across its inactive, intermediate, and active states. High-resolution atomic coordinates were retrieved from the RCSB Protein Data Bank (PDB), complemented by biochemical, kinetic, and post-translational modification data harvested from peer-reviewed literature across PubMed and Scopus up to 2026. Analytical strategies involved sequence-to-structure mapping using PyMOL 3.1 to define the precise topological boundaries of the PYD, LRR, and five segmented NACHT subdomains (FISNA, NBD, HD1, WHD, HD2).

**Figure 1:**
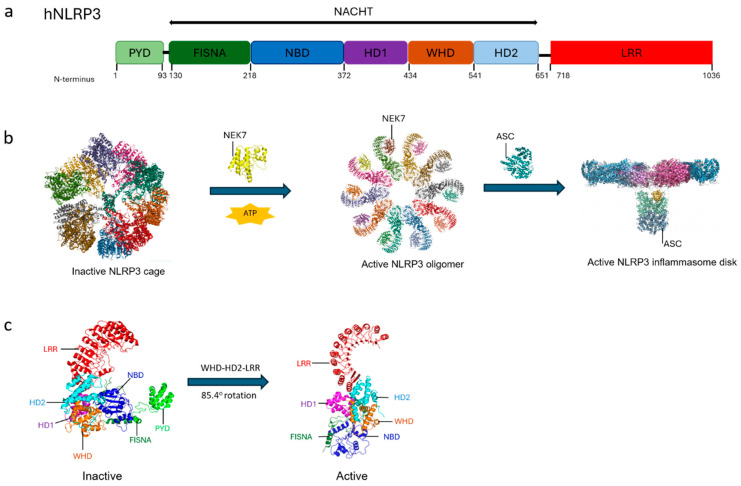
Structural architecture of NLRP3 and the molecular mechanism of NLRP3 inflammasome activation. (**a**) Schematic domain blueprint of human full-length NLRP3, depicting the N-terminal pyrin domain (PYD, green), the central catalytic NACHT domain comprising the fish-specific NACHT-associated domain (FISNA, dark green), nucleotide-binding domain (NBD, blue), helical domain 1 (HD1, purple), winged helix domain (WHD, orange), and helical domain 2 (HD2, light blue), and the C-terminal Leucine-Rich Repeat domain (LRR, red). (**b**) Proposed multi-step model for high-order assembly and structural transition of the NLRP3 inflammasome. In the resting state, inactive ADP-bound NLRP3 forms a closed, self-inhibited double-ring oligomeric cage (left; PDB ID: 7PZC) that sequesters the PYD domains inside its hollow internal cavity. Upon sensing danger signals, nucleotide exchange (ADP to ATP) and the chaperone-like binding of the mitotic kinase NEK7 (yellow) to the LRR motifs trigger the disassembly of the cage into open, flower-shaped, inflammasome-competent NLRP3 complexes (middle; PDB ID: 8EJ4). These complexes subsequently recruit the adaptor protein ASC (teal) to form the functional, macromolecular disk-like core of the active NLRP3 inflammasome (right; PDB ID: 8ERT). (**c**) Detailed overlay highlighting the large-scale conformational rearrangements within a single NLRP3 NACHT domain during the transition from the inactive (ADP-bound) to active (ATP-bound) status. The structural reorganization is driven by a pronounced 85.4° rigid-body rotation of the WHD-HD2-LRR module relative to the NBD-HD1 core, opening the catalytic domain to expose interfaces required for downstream oligomerization and filament recruitment.

A molecular-level understanding of the NLRP3 inflammasome activation mechanism will provide deep insights into inhibition pathways for drug development. Structurally, the NLRP3 protein consists of three distinct domains, including the pyrin domain (PYD) (residues 1 to 93), NACHT domain (residues 130 to 651) and Leucine-Rich Repeat domain (LRR) (residues 718 to 1036). The central catalytic NACHT domain comprises a fish-specific NACHT-associated domain (FISNA) (residues 130 to 218), a nucleotide-binding domain (NBD) (residues 218–372), a helical domain 1 (HD1) (residues 372–434), a winged helix domain (WHD) (residues 434–541), and a helical domain 2 (HD2) (residues 541–651) (Figure 1a,c) [24,25]. The NBD subdomain features Walker A and Walker B sites. Walker A binds ATP to trigger the conformational shift from an autoinhibited to an active, open state, though it binds ADP in its resting form. Meanwhile, Walker B coordinates the magnesium ion (Mg^2+^) necessary for ATP hydrolysis. Recent structural biology studies show full-length inactive NLRP3 in the presence of ADP forms large, high-order, double-ring oligomeric assemblies referred to as “cages” or “barrels”; these are primarily 12- to 16-mer cages in mice and decameric cages in humans. These closed configurations are structurally driven and locked by LRR–LRR interactions (Back–Back and Face–Face), while the PYD domains are hidden inside the hollow internal cavity of the assembly, enforcing an autoinhibited state (Figure 1b) [26,27].

Crucially, NLRP3 activation is a sophisticated, multi-layered regulatory event where nucleotide exchange alone is insufficient to drive the full cascade. Under basal conditions, NLRP3 is heavily regulated by an array of post-translational modifications (PTMs), such as baseline phosphorylation and ubiquitination, which maintain the protein in a licensed but inactive state. Upon sensing diverse danger signals, most notably intracellular K^+^ efflux, downstream cascades are triggered that often induce changes in the subcellular localization of NLRP3, prompting its translocation to distinct membranes such as the trans-Golgi network (TGN) or mitochondria [28]. This altered microenvironment facilitates structural remodeling and licenses the NACHT domain for nucleotide exchange.

Activation of NLRP3 requires nucleotide (ATP) binding to NACHT domain, followed by conformational changes in the FISNA domain. The WHD-HD2-LRR region undergoes a rigid body rotation of about 85.4° from its position in the closed conformation (Figure 1c) [27,29,30]. Functional studies show that the kinase NEK7 is crucial for NLRP3 activation in macrophages [31,32,33]. Cryo-EM structural analysis of active NLRP3 reveals that mitotic kinase NEK7 can bind to LRR motifs of NLRP3 and act as an NLRP3 chaperone in its transition from an ADP-bound inactive conformation to the ATP-bound active conformation without contributing to the stabilization of the active NLRP3 inflammasome complex (Figure 1b) [34]. Another interesting observation is that the presence of NEK7 can lead to the dissociation of large NLRP3 oligomers from both closed and open conformations into NEK7/NLRP3 dimers, which are critical for assembly of the disk-like inflammasome [35]. Subsequently, the open NEK7/NLRP3 allows for uni- or bidirectional inflammasome assembly and recruits ASC to initiate the formation of the hybrid PYD-PYD filament between NLRP3 and ASC [35]. The adaptor ASC is linked with caspase-1 through CARD-CARD interactions to form speck-like aggregates (Figure 1b). Caspase-1 catalytic domains dimerize to become activated, then proteolytically mediate GSDMD activation and cytokine maturation [6,7].

## 3. NLRP3 Inhibitors

Elucidating the molecular mechanisms of NLRP3 activation and inhibitor binding modes is crucial for accelerating drug design. This review summarizes existing NLRP3 inhibitors characterized by cryo-EM/X-ray structural studies.

### 3.1. MCC950 and Analogs of Sulfonylureas

**Figure 2:**
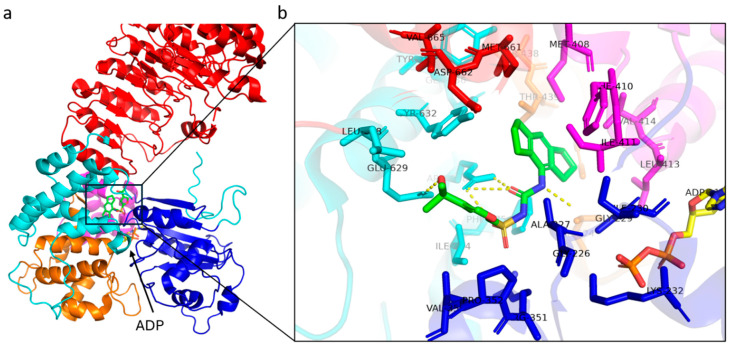
Overall binding architecture and detailed molecular interactions of MCC950 within the human NLRP3 NACHT domain. (**a**) Overview of the allosteric binding model of the prototypical inhibitor MCC950 (represented as green sticks) complexed with the inactive human NLRP3 decamer (PDB ID: 7PZC). (**b**) Close-up view of the explicit atomic interactions stabilizing MCC950 at the interface of the NACHT subdomains.

Researchers at Pfizer found that sulfonylurea compounds can inhibit IL-1β release. One of compounds, MCC950, also known as CP-456,773 or CRID3, was later confirmed as a remarkably potent and selective inhibitor of the NLRP3 inflammasome [22]. Cryo-electron microscopy structures of the full-length human NLRP3 decamer in its native form and complexed with the inhibitor MCC950 revealed its binding mode. The compound occupies an allosteric package formed by subdomains NBD, HD1, WHD, HD2 and LRR near the nucleotide-binding site (Figure 2a) [27]. Mechanistically, the tricyclic hexahydro-s-indacene moiety of MCC950 formed multiple hydrophobic contacts with the residues GLY229 (NBD), MET408, PHE410, ILE411, LEU413, V414 (HD1), THR439, TYR443, THR524 (WHD), PHE575, ARG578, TYR632 (HD2), and MET661 (LRR). The tricyclic sulfonyl amide moiety mainly forms a hydrogen bond with ARG578 (HD2) and an ionic interaction with ARG351 (NBD). The propanol moiety, positioned close to the solvent-exposed area, forms an additional hydrogen bond with GLU629 (HD2) (Figure 2b). The binding package is mediated by NACHT and LRR in closed form and clearly demonstrates that MCC950 inhibits the opening of NACHT for oligomerization, maintaining its inactive, nucleotide-bound form to prevent the ATP-dependent conformational cycling, oligomerization of NLRP3, and formation of the inflammasome assembly [27].

**Figure 3:**
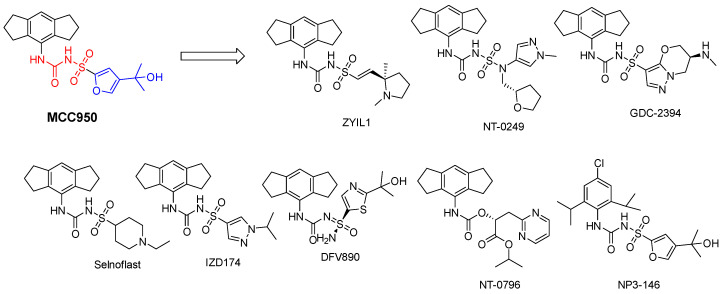
Chemical evolution and structural diversity of NLRP3 inhibitors derived from MCC950.

The confirmation of MCC950 as a direct NLRP3 inhibitor has initiated the first wave of NLRP3 drug development, primarily based on the MCC950 sulfonylurea scaffold. Modifications were focused on the hexahydro-s-indacene moiety, sulfonylurea and furan propanol moiety, respectively. NP3-146 was designed by replacing the hexahydro-s-indacene moiety with 1-chloro-3,5-diisopropylbenzene (Figure 3) [29]. Several representative inhibitors have entered the clinical trial at different stages, including ZYIL-1, NT-0249, GDC-2394, IZD174, selnoflast and DFV890 (Figure 3) [36,37,38,39,40,41]. Variation in the furan propanol moiety involves chain extension (ZYIL1), spatial expansion (NT-0249, GDC-23394), and ring replacement (IZD174, selnoflast), while DFV890 and NT0796 were developed to explore the chiral preference of sulfonylurea moiety. The variety of modifications indicated the flexibility of each part of MCC950 in targeting the binding package.

**Figure 4:**
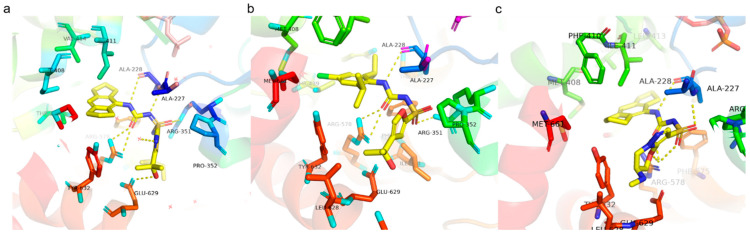
Comparative molecular docking landscapes and atomic interactions of structurally diverse MCC950 derivatives within the NLRP3 NACHT pocket. (**a**) Atomic interactions of the chiral sulfonimidamide-containing inhibitor DFV890 (PDB ID: 9HG4). (**b**) Structural analysis of the diaryl sulfonylurea analogue NP3-146 (PDB ID: 7ALV). (**c**) Binding conformation of the bulky, structurally reorganized clinical candidate GDC-2394 (PDB ID: 8ETR).

Crystal structures of the NLRP3 NACHT domain bound to GDC-2394 [42], DFV890 [43] and NP3-146 [29] reveal that modifications to the three regions of MCC950 are well-tolerated within the binding pocket, mimicking the binding mode of MCC950. The chiral sulfonimidamide core of DFV890 affords multi-hydrogen bonds with ALA-228, ALA-227, ARG578, and ARG-351, while the propanol moiety forms an additional hydrogen bond with GLU629 (Figure 4a). The 1-chloro-3,5-diisopropylbenzene moiety of NP3-146 occupies the same site as the hexahydro-s-indacene moiety of MCC950 to exhibit a hydrophobic effect, while other parts, like MCC950, form hydrogen bonds with ALA-228, ARG-578 and ARG-351 (Figure 4b). The sulfonylurea moiety of GDC-2394 can form hydrogen bonds with ALA-228, ALA-227, and ARG-578. Notably, the 6,7-dihydro-5H-pyrazolo[5,1-b][1,3]oxazine moiety of GDC-2394 is different from the furan propanol moiety, which can form an extra hydrogen bond with ARG-578 instead of GLU-629 (Figure 4c).

### 3.2. Oxazole Acylsulfamide NLRP3 Inhibitors

**Figure 5:**
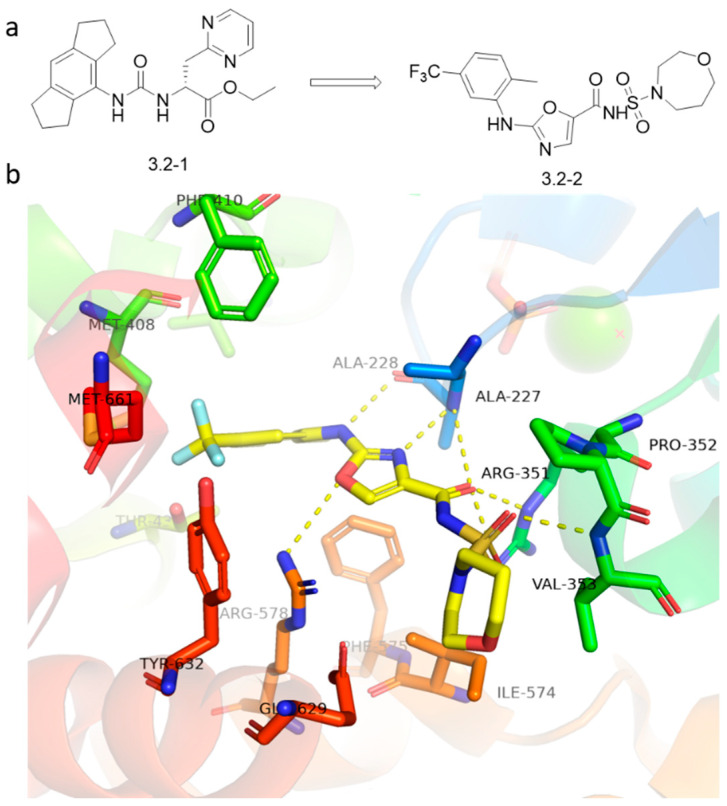
(**a**) Structures of hit compound 3.2-1 and candidate 3.2-2. (**b**) Atomic interactions between oxazole acylsulfamide NLRP3 inhibitor 3.2-2 and NLRP3 residues formed at the interface of the four NACHT subdomains (PDB: 8WSM).

Japan Tobacco Inc. focused on compound 3.2-1 and adopted a scaffold-hopping strategy to explore core motifs using a ring closing approach, after which an oxazole-based scaffold was identified. Subsequent implementation of a bioisosteric replacement led to the discovery of a novel chemical class of NLRP3 inflammasome inhibitors bearing the acylsulfamide group, exemplified by compound 3.2-2 (Figure 5a). The most potent compound showed an IC_50_ of 21 nM and 0.57 μM on the IL-1β release inhibition in THP-1 cells and in a whole blood assay, respectively [44]. The X-ray structure of compound 3.2-2 in the human NLRP3 NACHT domain revealed that the compound directly binds to the NACHT domain of human NLRP3. The NH bond of the aniline group and the nitrogen atom in the oxazole scaffold form a strong hydrogen bond with Ala228 and ALA-227. Meanwhile, the acylsulfamide group was found to form hydrogen bonds with residues ARG351 and VAL353 (Figure 5b). Overall, it shares a similar binding site with MCC950 but picks up different residues to form hydrogen bonds in the NLRP3 NACHT domain [29].

### 3.3. Tricyclic NLRP3 Inhibitors

**Figure 6:**
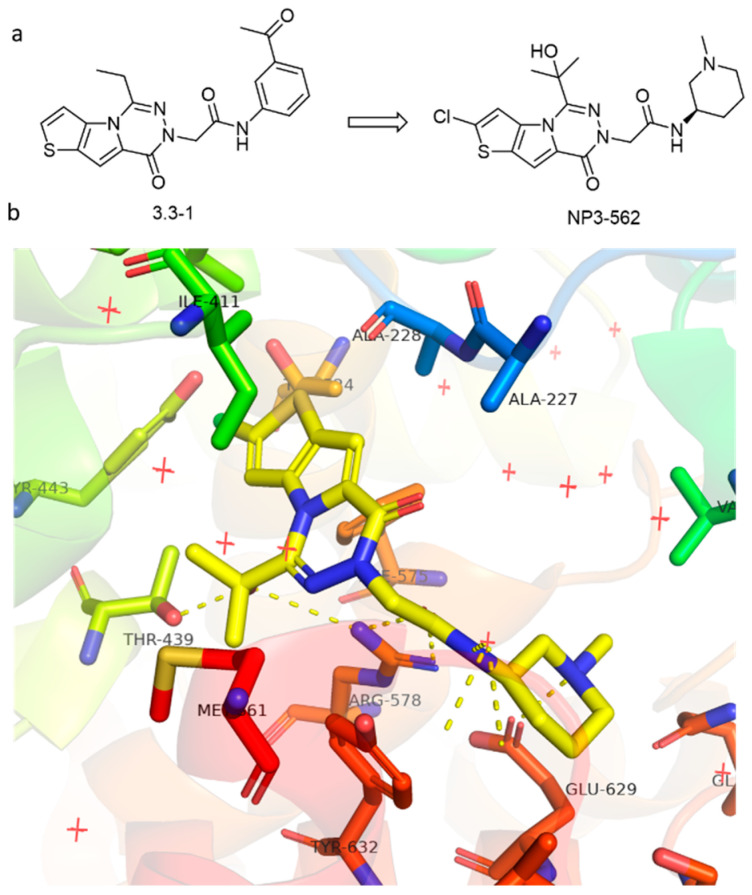
Identification, structural optimization, and atomic interactions of the novel oxazole acylsulfamide NLRP3 inhibitor series. (**a**) Chemical structures of the initial screening hit compound 3.3-1 and its optimized, advanced lead candidate NP3-562. (**b**) Detailed structural view of the atomic interactions between NP3-562 and key coordinating residues within the inactive human NLRP3 NACHT domain (PDB ID: 8WSM).

To expand the chemical landscape of NLRP3 inhibitors beyond the known sulfonylurea-based scaffold, a high-throughput screen was conducted by Novartis utilizing pyroptosis induced by LPS/nigericin in PMA-differentiated THP-1 cells against their compound collection [45]. Orthogonal readout of IL-1β inhibition, a TNF-α counter screen and direct binding effects on NLRP3 measured through the previously described biochemical fluorescence polarization (FP) assay [29] identified hit 3.3-1. It showed an IC_50_ of 0.63 μM on inhibition of IL-1β release from nigericin-stimulated THP-1 and an IC_50_ of 1.13 μM in FP assay without effect on THF-α release (IC_50_ > 100 μM). Structure–Activity Relationship (SAR) analysis enabled the discovery of NP3-562 (Figure 6a). The compound was co-crystallized with the NLRP3 NACHT domain (PDB: 8RI2), revealing the same ligand binding site as MCC950, which is located at the interface of four subdomains, i.e., NBD, HD1, WHD and HD2. In comparison, the crystal structure revealed that the tricycle of NP3-562 occupies a longer, narrower hydrophobic pocket than the tricyclic hexahydro-s-indacene moiety of MCC950, interacting with lipophilic residues including Ala227, Ala228, Met408, Ile411, Leu413, and Val414. This feature enables Arg578 and Thr439 to contact the ligand by interacting with its alcohol substituent. Closer to the solvent-exposed region, key hydrogen-bonding interactions were observed between the amide carbonyl and Arg578, as well as between the piperidine nitrogen and Glu629. In addition, a water-mediated interaction between the NH and the Asp662 residue illustrates the importance of the ligand’s secondary amide (Figure 6b) [46].

**Figure 7:**
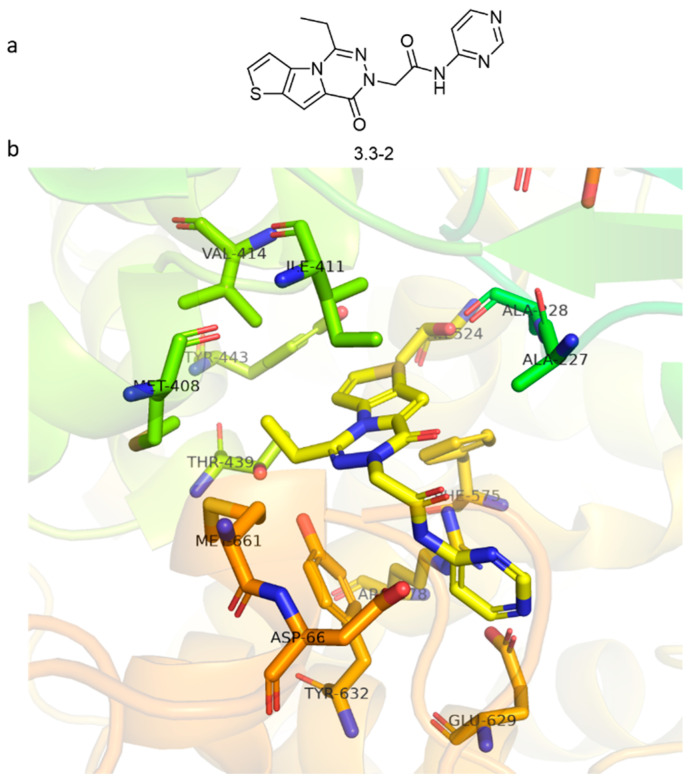
(**a**) Structures of compound 3.3-2. (**b**) Detailed close-up view of the explicit atomic interactions between compound 3.3-2 and the coordinating amino acid residues within the human NLRP3 NACHT domain (PDB ID: 9DH3).

Another similar scaffold was reported by Janssen LLC. To identify selective NLRP3 inflammasome inhibitors, a phenotypic screening funnel was established beginning with a primary assay utilizing LPS-primed, nigericin-activated murine J774A.1 macrophages, with cell death serving as the primary readout. Confirmed hits were then subjected to a counter screen against an NLRC4-selective inflammasome activator to eliminate off-target compounds and ensure NLRP3 specificity. Finally, downstream validation was conducted using human peripheral blood mononuclear cells (PBMCs); these were stimulated with LPS for 6 h, allowing for the isolation of potent, selective inhibitors of IL-1β secretion that left general, NF-κB-mediated cytokines (IL-6 and TNF) unaffected. Target engagement was performed with Nano-differential scanning fluorometry (DSF) with recombinant MBP-hNLRP3-ΔPYD protein. The cryo-EM structure (PDB: 9DH3) of MBPhNLRP3-ΔPYD in complex with ADP and compound 3.3-2 formed a tetramer via the MBP-NACHT and back–back (between LRR and LRR) interactions with D2 symmetry (Figure 7a,b). Compound 3.3-2, similar to NP3-562, adopts a closed pose. Structurally, compound 3.3-2 buries its tricyclic moieties in a hydrophobic pocket and extends its respective outer groups and pyrimidine toward the LRR region. The amide linkers anchor Arg578 and Asp662. However, the removal of the alcohol substituent occurs as a result of the H-bond interaction between Arg578 and Thr439 residues. Furthermore, no obvious hydrogen bond formation is observable within the structure (Figure 7b) [47].

### 3.4. 3-Amino-6-phenyl-pyridazine-Based NLRP3 Inhibitors

**Figure 8:**
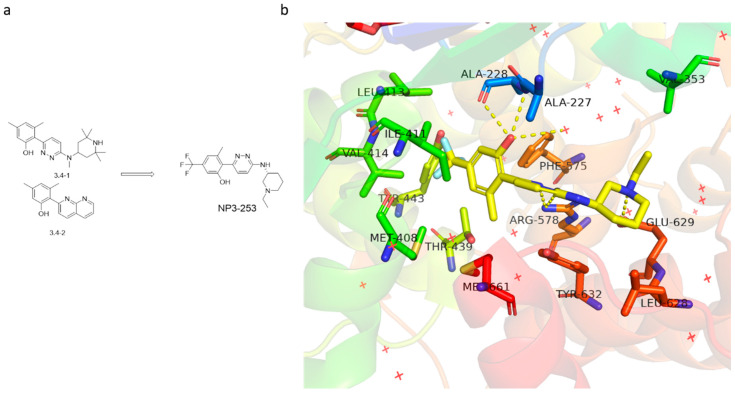
(**a**) Structures of hit compounds 3.4-1-2 and NP3-253. (**b**) Atomic interactions between NP3-253 and NLRP3 residues formed at the interface of the four NACHT subdomains (PDB: 9GU4).

Using the same screening procedure mentioned in Section 3.3, Novartis found another chemotype of hit 3.4-1 and 3.4-2 (Figure 8a). Their medicinal chemistry program guided the discovery of the new class of 3-amino-6-phenyl-pyridazine chemotype NLRP3 inhibitors, exemplified by NP3-253 [48]. Using the NACHT domain [29], crystallization with NP3-253 indicated that this chemical entity also aligned in the same pocket as NP3-562. The phenol occupies a hydrophobic cavity against Ile411, Thr439, and Tyr443. Residue Ala228 in the NBD can form a H-bonding interaction with OH. A key bidentate interaction is established between the pyridazine core and the Arg578 side chain. The piperidine nitrogen forms a salt bridge with Glu629, which additionally bridges to Arg578. This cooperative network mutually stabilizes the Arg578–pyridazine and Glu629–piperidine amine interactions. Overall, the binding site of NP3-253 is similar to that of NP3-562, in which phenol and tricycle occupy the same hydrophobic cavity, while pyridazine nitrogens play the same role as the carbonyl oxygen of NP3-562 with Arg578. The rotational flexibility between the phenol and pyridazine of NP3-253 allows for the optimal interaction of pyridazine with Arg578 and Glu629 (Figure 8b). This binding mode involves four subdomains of the NACHT domain and stabilizes in a closed conformation to inhibit conformational changes. This new development paved the way for a second wave of brain-penetrant NLRP3 inhibitors.

**Figure 9:**
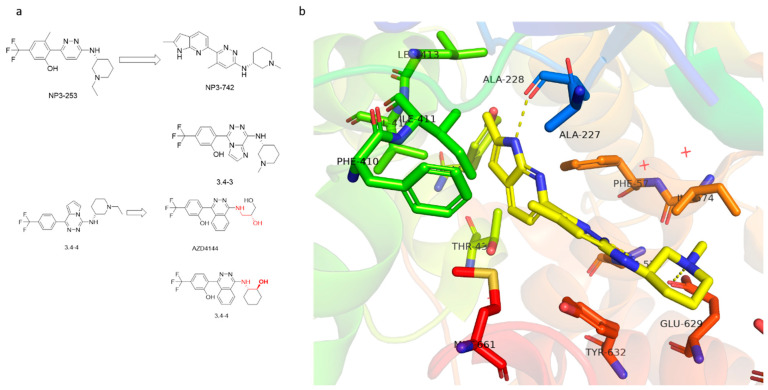
(**a**) Structures of representative compounds derived from NP3-253. (**b**) Atomic interactions between NP3-742 and NLRP3 residues formed at the interface of the four NACHT subdomains (PDB: 9SFG).

Numerous structural modifications have been made to NP3-253, focusing specifically on three moieties: the phenyl, pyridazine, and piperidine rings. A comprehensive summary of these changes can be found in our previous review [49]. The representative compounds are shown in Figure 9a. Replacement of the phenol with an indole group yields NP3-742 [50]. The X-ray crystal structure of compound NP3-742 bound to the NLRP3 NACHT domain (PDB: 9SFG) reveals that it adopts a binding mode similar to that of NP3-253. The central pyridazine ring forms a dual-hydrogen-bond-mediated interaction with Arg578, while the piperidine nitrogen engages in interaction with Glu629. In comparison to the phenol in NP3-253, the indole ring occupies a deep lipophilic pocket and the methyl group on the pyridazine ring fills a small hydrophobic pocket. The indole-NH enables a hydrogen bond donation by Ala-228 similar to that of the phenol (Figure 9b) [48]. To balance between brain penetration and hERG inhibition, Janssen LLC focused efforts toward NP3-253 analogues containing 5,6-bicyclic cores [51]. The discovery of compound 3.4-3 and the binding mode of the methyl group in NP3-742 suggests that modifying the pyridazine ring could exploit the hydrophobic effect within this binding pocket. The BioPharmaceuticals R&D also disclosed hit 3.4-4 through a high throughput assay using lipopolysaccharide (LPS)-stimulated THP-1 cells under treatment with benzoyl-ATP(BzATP) to trigger IL-1β release. Optimization of 3.4-4 led to the discovery of AZD4144, a valuable prototype for exploring replacements of the piperidine moiety. Within this structural class, the NH-C_2_H_4_-OH and NH-C_2_H_4_-NR_2_ motifs are preferred (Figure 9a) [52].

### 3.5. CNS-Penetrating Indazole Scaffold

**Figure 10:**
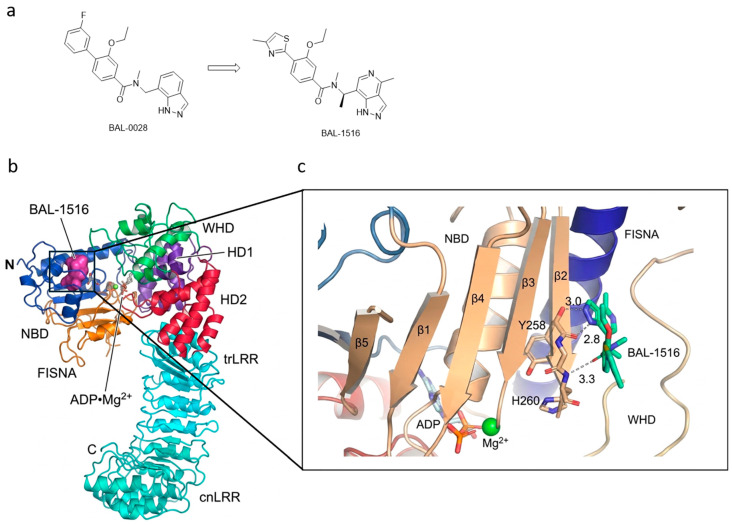
Chemical structures and structural model of the novel indazole-based NLRP3 inhibitor series. (**a**) Chemical structures of the screening lead BAL-0028 and its optimized, brain-penetrant analogue BAL-1516. These compounds represent a structurally distinct indazole chemotype discovered via DNA-encoded library (DEL) screening. (**b**) Structural model detailing the unique binding modality of BAL-1516 within human NLRP3. (**c**) Atomic interactions between BAL-1516 and NLRP3 residues formed at the interface of NBD, WHD and FISNA. While experimental cryo-EM data confirm that BAL-1516 binds to the inactive decameric assembly of NLRP3, the coordinates are currently unreleased in the Protein Data Bank (PDB). Therefore, this panel illustrates a high-confidence structural model generated based on the published literature to show how BAL-1516 targets a surface-accessible groove at the junction of the FISNA, NBD, and WHD subdomains. Key interactions match the experimentally reported main-chain hydrogen bonds established with the backbone carbonyl motifs of Tyr258 and His260.

BioAge Labs utilize DNA-encoded library screening against IL-1β inhibition in PMA-differentiated THP-1 under LPS/nigericin conditions, which have allowed for the discovery of hit BAL-0028 [53]. Further optimization of BAL-0028 yielded BAL-1516 (Figure 10a). The cryo-EM structure of the NLRP3-BAL-1516 decamer was gained from the expression of human full-length NLRP3 purification in the presence of BAL-1516. The binding site is distinct from those of other types of compounds, which were formed by subdomains of NBD, WHD and FISNA (Figure 10b). The hydrophobic thiazole–phenyl ring system packs against the central *β*-sheet of the NBD from behind the ADP nucleotide. The ethoxy group at the para position of the phenyl ring occupies a hydrophobic core. This portion largely exhibits hydrophobic effects. The chiral center of BAL-1516 induces a distinct kink between the indazole and the methyl–aminocarbonyl groups; the carbonyl forms a H-bond with Glu263, and the methyl-mediated (*R*)-configuration of the indazole fits precisely into the pocket. Three hydrogen bonds are formed between the 5-azaindazole ring, the aminocarbonyl linker of the compound and the main-chain atoms of Tyr258 and the His260 B2 strand (Figure 10c) [54].

### 3.6. Summary of Direct NLRP3 Inhibitors and Binding Mode

The structural elucidation of various small-molecule inhibitors reveals that while most direct NLRP3 antagonists converge on the central NACHT domain, they exploit distinct binding landscapes, specific amino acid networks, and structural dynamics that dictate their therapeutic potential. Traditional sulfonylureas (such as MCC950), alongside newer oxazole acylsulfamides (e.g., compound 3.2-2), tricyclics (e.g., NP3-562), and phenol–pyridazines (e.g., NP3-253), predominantly target a highly conserved, deeply buried allosteric pocket at the interface of the NBD, HD1, WHD, HD2, and LRR subdomains. Within this pocket, these structurally diverse scaffolds anchor themselves through shared key interactions, establishing crucial hydrogen bonds or salt bridges with Arg578 and Glu629, or forming hydrogen bonds with Ala227 and Ala228. Overall, the exact spatial tolerances and mechanical effects within this shared cavity are highly scaffold-dependent. These scaffolds commonly feature a bulky tricyclic or block core that anchors deeply within the cavity via hydrophobic effects. Their central motifs (sulfonylureas, amides, or pyridazines) establish essential hydrogen bonds with pocket residues, while solvent-exposed peripheral groups, such as piperidine and furan-propanol, provide additional stabilization through mixed hydrophobic and hydrogen-bonding interactions. Notably, the novel indazole-based chemotype BAL-1516 completely diverges by occupying an entirely distinct surface groove behind the nucleotide-binding pocket formed by the FISNA, NBD, and WHD subdomains, utilizing main-chain hydrogen bonds with Tyr258 and His260. Functionally, both binding modalities successfully lock the flexible NACHT domain into an inactive, closed conformation (Table 1). Targeting the traditional MCC950 pocket historically limits chemical diversity and leaves compounds vulnerable to drug resistance from hereditary NACHT mutations. Conversely, alternative surface grooves like the BAL-1516 site offer an excellent blueprint for mutation-agnostic strategies, though structure-based design remains constrained by a relative scarcity of resolved structures.

## 4. Challenges and Future Directions

The timeline of NLRP3 inhibitor development spans over two decades, anchored by the discovery of MCC950 as the foundational, first-in-class direct NLRP3 inhibitor [27]. For years, medicinal chemistry efforts heavily prioritized structural modifications based on this specific scaffold, which subsequently led to the identification of several chemical classes. Later, phenol–pyridazine-based NLRP3 inhibitors initiated a second wave of brain-penetrant drug development [55]. However, many of these early hits were discovered using phenotypic screening methods. Although valuable, phenotypic assays risk yielding false-positive or off-target results due to their reliance on complex downstream endpoints like IL-1β inhibition. Additionally, assay potency can vary across species. These endpoints are influenced by an array of intersecting pathways, including NF-kb signaling, ionic fluxes (such as potassium efflux and calcium influx), reactive oxygen species (ROS) production, mitochondrial dysfunction, and downstream inflammasome cascades involving caspase-1 activation and gasdermin D (GSDMD) cleavage [2]. Furthermore, when validating whether these phenotypic hits directly bound NLRP3, researchers frequently relied on MCC950-derived competitive displacement tools, such as fluorescence polarization (FP) [29], target engagement assays [56] and scintillation proximity assays (SPAs) [57]. Consequently, this biased the discovery process toward hits that shared the exact same binding pocket or mechanism of action as MCC950, severely limiting the chemical and biological diversity needed to overcome clinical hurdles. To bypass these limitations, there is an urgent need to establish robust, direct-to-target biochemical assays. Recent crystallization studies have illuminated the structural landscape of the NLRP3 NACHT domain, revealing two distinct binding sites: an allosteric pocket (MCC950 site) and a surface groove of nucleotide-binding domain (BAL-1516 site). While these structural insights provide a rational blueprint for designing novel inhibitors, targeting other regions remains challenging. For instance, although small molecules targeting the canonical ATP/ADP-binding site have been reported, the lack of resolved crystal structures for this specific configuration hinders structure-based drug design. Moreover, inhibitors targeting the leucine-rich repeat (LRR) or pyrin (PYD) domains, as well as protein–protein interaction (PPI) disruptors, remain exceedingly rare. Thus, despite significant preclinical milestones in NLRP3 inhibitor development, diversifying the structural targets beyond the traditional MCC950 paradigm represents the next critical frontier. The primary challenge in deploying small-molecule NLRP3 inhibitors for hereditary human mutations is overcoming structural heterogeneity and resistance. Because over 100 distinct gain-of-function mutations exist, largely clustering in the NACHT domain, a single inhibitor may effectively silence one variant while failing completely against another that has a structurally altered drug-binding pocket. Furthermore, mutated NLRP3 proteins are often locked into an energetically favorable, rigidly active conformation, requiring exponentially higher drug potencies to disrupt them compared to unmutated proteins. To overcome these hurdles, the paradigm of NLRP3 inhibitor development is shifting toward mutation-agnostic strategies. Rather than attempting to block the highly variable, mutation-prone NACHT (ATPase) domain where traditional molecules like MCC950 bind, modern drug discovery focuses on targeting the invariant, structurally conserved checkpoints required for inflammasome assembly.

## 5. Conclusions

NLRP3 has emerged as a highly promising therapeutic target for a diverse range of inflammatory diseases. The development of direct NLRP3 inhibitors represents a paradigm shift in modern drug discovery. In this review, we first elucidated the structural biology underlying NLRP3 activation mechanisms, highlighting key insights that enable researchers to decipher how small molecules can disrupt these pathways. Next, we provided a chronological overview of drug development, tracing the journey from initial hit screening to candidate optimization. Specifically, we categorized and systematically discussed the molecular-level binding modes of key chemical scaffolds, including sulfonylureas, oxazole acylsulfamides, tricyclics, phenol–pyridazines, and indazoles. By mapping the current landscape of direct NLRP3 inhibitors and providing a rigorous mechanistic analysis at the molecular level, this review aims to facilitate the rational design and continued development of next-generation, NLRP3-targeted therapeutics.

## Figures and Tables

**Table 1:** Summary of direct NLRP3 inhibitors.

Compounds in Red Indicate H-Bonding Sites	Pocket (NACHT Domain of NLRP3)	Residues for H-Bond	PDB	Potency (IL-1 β Inhibition)	Ref.
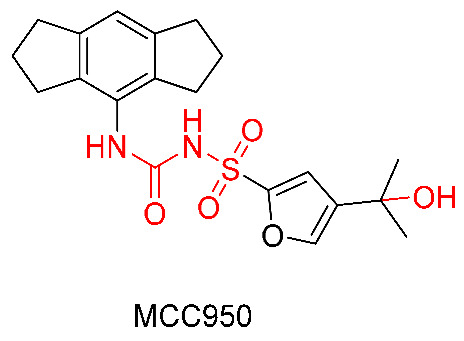	Allosteric package formed by subdomains NBD, HD1, WHD, HD2 and LRR	ALA227 ARG578 GLU629	7PZC	BMDMs IC_50_: 7.5 nM THP-1 IC_50_: 10–15 nM	[22,27]
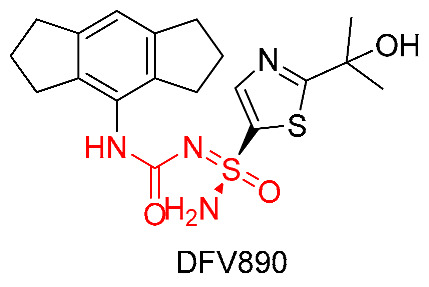	Allosteric package formed by subdomains NBD, HD1, WHD, HD2 and LRR	ALA-228, ALA-227, ARG578, ARG-351, GLU629	9HG4	THP-1, and PBMC IC_50_: 1–2.9 nM	[36,43]
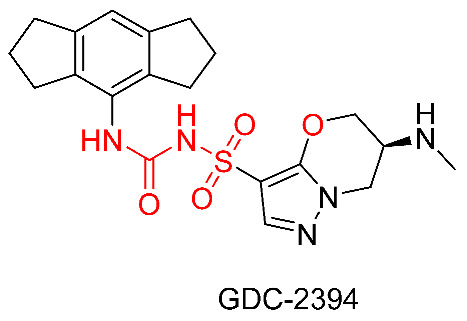	Allosteric package formed by subdomains NBD, HD1, WHD, HD2 and LRR	ALA-228, ALA-227, ARG-578	8ETR	PMBC IC_50_: 5.4 nM	[41,42]
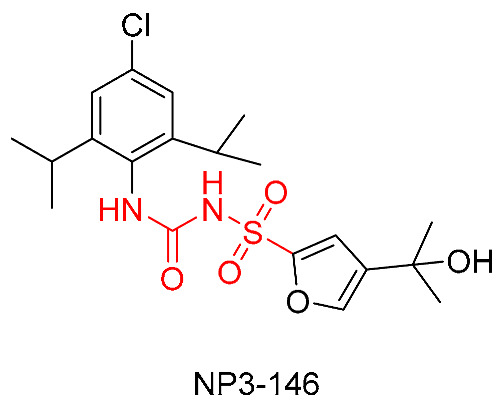	Allosteric package formed by subdomains NBD, HD1, WHD, HD2 and LRR	ALA-228, ARG-578, ARG-351	7ALV	BMDM IC_50_: 0.171 μM	[29]
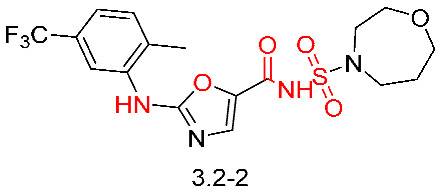	Allosteric package formed by subdomains NBD, HD1, WHD, HD2 and LRR	ALA228, ALA-227, ARG351, VAL353	8WSM	THP-1 IC_50_: 21 nM	[44]
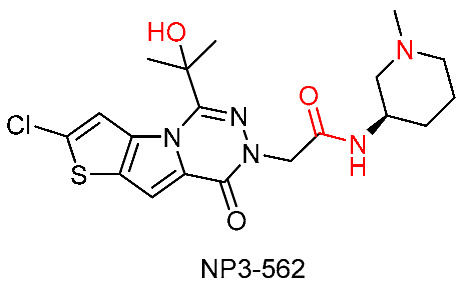	Allosteric package formed by subdomains NBD, HD1, WHD, HD2 and LRR	Arg578, Glu629, Asp662	8RI2	THP-1 IC_50_: 90 nM	[46]
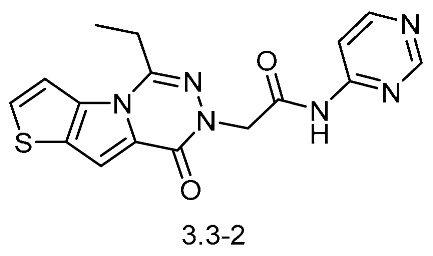	Allosteric package formed by subdomains NBD, HD1, WHD, HD2 and LRR	NA	9DH3	PBMCs IC_50_: 41 nM	[47]
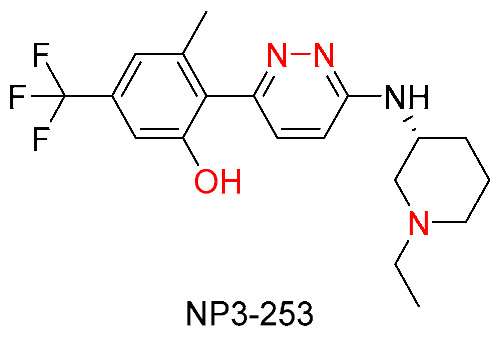	Allosteric package formed by subdomains NBD, HD1, WHD, HD2 and LRR	Ala228, Arg578, Glu629	9GU4	THP-1 IC_50_: 0.5 nM	[48]
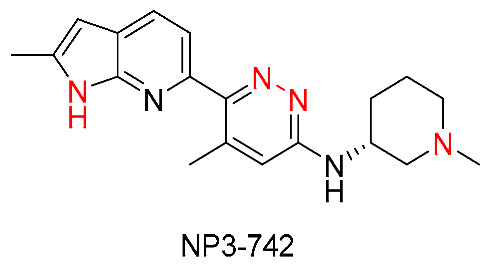	Allosteric package formed by subdomains NBD, HD1, WHD, HD2 and LRR	Ala228, Arg578, Glu629	9SFG	THP-1 IC_50_: 6 nM	[50]
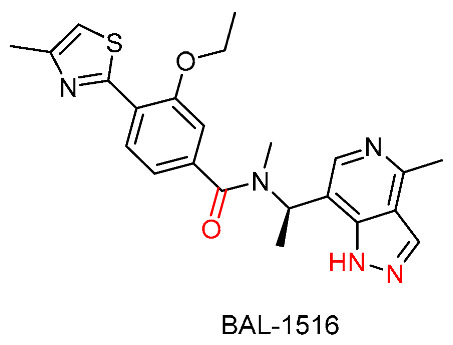	Surface groove formed by FISNA, NBD and WHD	Glu263, Tyr258, His260	NA	Human iPSC-derived microglia and microglial IC_50_: 10–15 nM	[53]

## Data Availability

No new data were created or analyzed in this study. Data sharing is not applicable to this article.

## Abbreviations

The following abbreviations are used in this manuscript:

NLRP3	NOD-like receptor family pyrin domain containing 3
Cryo-EM	Cryo-electron microscopy
DAMPs	Damage-associated molecular patterns
PAMPs	Pathogen-associated molecular patterns
GSDMD	Gasdermin D
ASC	Apoptosis-associated speck-like protein
IL-1β	Interleukin-1 beta
PYD	Pyrin domain
LRR	Leucine-Rich Repeat Domain
FISNA	Fish-specific NACHT-associated domain
NBD	Nucleotide-Binding Domain
HD1	Helical domain 1
WHD	Winged helix domain
HD2	Helical domain 2
FP	Fluorescence polarization
DSF	Nano-differential scanning fluorometry
LPS	Lipopolysaccharide
BzATP	Benzoyl-ATP
PBMCs	Peripheral blood mononuclear cells
